# Adaptive management of energy consumption, reliability and delay of wireless sensor node: Application to IEEE 802.15.4 wireless sensor node

**DOI:** 10.1371/journal.pone.0172336

**Published:** 2017-02-24

**Authors:** Cheick Tidjane Kone, Jean-Denis Mathias, Gil De Sousa

**Affiliations:** 1 DRIT, Esatic, Abidjan, Côte d’Ivoire; 2 UR LISC, Irstea, Aubière, France; 3 UR TSCF, Irstea, Aubière, France; Tianjin University of Technology, CHINA

## Abstract

Designing a Wireless Sensor Network (WSN) to achieve a high Quality of Service (QoS) (network performance and durability) is a challenging problem. We address it by focusing on the performance of the 802.15.4 communication protocol because the IEEE 802.15.4 Standard is actually considered as one of the reference technologies in WSNs. In this paper, we propose to control the sustainable use of resources (i.e., energy consumption, reliability and timely packet transmission) of a wireless sensor node equipped with photovoltaic cells by an adaptive tuning not only of the MAC (Medium Access Control) parameters but also of the sampling frequency of the node. To do this, we use one of the existing control approaches, namely the viability theory, which aims to preserve the functions and the controls of a dynamic system in a set of desirable states. So, an analytical model, describing the evolution over time of nodal resources, is derived and used by a viability algorithm for the adaptive tuning of the IEEE 802.15.4 MAC protocol. The simulation analysis shows that our solution allows ensuring indefinitely, in the absence of hardware failure, the operations (lifetime duration, reliability and timely packet transmission) of an 802.15.4 WSN and one can temporarily increase the sampling frequency of the node beyond the regular sampling one. This latter brings advantages for agricultural and environmental applications such as precision agriculture, flood or fire prevention. Main results show that our current approach enable to send more information when critical events occur without the node runs out of energy. Finally, we argue that our approach is generic and can be applied to other types of WSN.

## 1 Introduction

Agricultural and Environmental monitoring applications are essential for society. Generally in this type of application, the system must be able to provide quickly, over long time periods (from 1 to 5 years depending on the characteristics of the application) without human intervention, information about physical phenomena occurring in its close environment [1]. Today, Wireless Sensor Network (WSN) is used as an automatic data acquisition and transmission system in several agricultural monitoring applications. Indeed, the WSNs are autonomous systems at low cost, which do not necessarily require a pre-existing infrastructure. In addition, they are easy to deploy, even on isolated monitoring areas with impossible or difficult human being access. However, this type of network is composed of nodes called wireless sensor nodes, which usually have limited resources in terms of power transmission, processing capacity, collected data storage and energy. These inherent constraints have influenced many research issues in the field. For some kinds of wireless sensor platforms, data storage limitation can be overpassed by the use of a flash memory support such as a microSD card. However, energy constraint is still important for most of the WSN platforms. Indeed, the energy efficiency, by extension the network lifetime, is a major concern in the case of a deployment in inaccessible monitoring areas where it is often impossible to recharge or replace batteries of the wireless sensor nodes after depletion. This can be problematic in term of energy conservation especially for long duration applications as those of cacao crops where the information gathering takes about two years (i.e the harvest duration). The system must be self-energized given the long duration of the harvest. So, a smart and sustainable management of these resources is essential to meet the lifetime of WSN and the quality of service required by the application. Despite an energy supply from external sources (e.g., by solar panel), consumption management in WSN may still be problematic. For example, energy produced by a “renewable” source as a solar panel may be consumed more rapidly than it is produced, especially in Nordic countries with low solar radiation [2]; electricity generated by a solar photovoltaic (PV) system (i.e., total PV area and battery capacity) depends especially on variable meteorological conditions such as solar radiation, ambient temperature and wing speed. Differences in solar radiation levels also exist in continental climate areas between summer and winter seasons. In addition, the higher the energy efficiency is, higher will be the packet delay, and therefore does not meet the requirements of low-delay applications (e.g., emergency applications). In addition, the inherent hardware constraints (bandwidth, storage and memory capacities) of a wireless sensor node can cause large latencies and high packet loss and therefore a low level of communication Quality of Service (QoS) if the network density or the traffic is high. Classically, the network density corresponds to the number of nodes per surface unit. In a star topology network, it represents the maximum number of nodes that compete among themselves in order to communicate with the gateway or “sink node”. For example, in order to regulate more closely the crop needs in pesticides, fertilizers and irrigation, agriculture application may demand a high collection of information (soil and/or the growth of plants), i.e., a high sampling frequency. This may cause high latencies and packet losses resulting in low level of QoS. Thus, designing such network and, more specifically, the protocols to achieve high QoS (network performance and durability) is a challenging task.

Many WSN management schemes have been proposed to meet this issue. Most contributions have focused on possible network architecture and/or the development of software components (operating systems [3, 4], network layer [5–7], MAC layer [8–14] and PHY layer [15]) adapted to the limited resources of sensor nodes. However, this solution may cause major problems (e.g., failure handling, synchronization and inter-node communication) if the policies of management resources associated with each software component are very different and even opposed, especially in the heterogenous WSNs.

For improving the network performance, it is important to use a common method for the whole WSN to manage its energy resources. It is the goal of our approach that is different and complementary to those proposed in the scientific literature. The network performance can be evaluated through different metrics such as the robustness, the similarity [16] or complexity. In our study, we choose to evaluate the network performance through its reliability and packet delays. More precisely, we specify, at the start of each simulation, a required QoS based on a minimum reliability and maximum delay. Using this network performance criteria, the study focuses on how the viability theory operates, using its associated controls, in order to achieve or not this required QoS level. We propose in this paper to control the sustainable use of resources (i.e., energy consumption) of a wireless sensor node by an adaptive tuning of the MAC parameters (*macMinBE*, *macMaxCSMABackoffs* and *macMaxFrameRetries*). We also propose to adjust the offered load per node with the parameter (*λ*) of the sampling frequency in order to meet, in real-time, agricultural and environmental application requirements. In these application topics, measurements can be collected at different frequencies in order to determine, for example, optimal quantities of agricultural inputs (fertilizers, irrigation, pesticides, etc.). For example, when the temperature in a forest increases roughly, the node could benefit of this (viability evaluated) “extra energy” to provide critical information about these environmental phenomenum at higher frequencies and, after, it could come back to a normal activity state and continue its tasks. In many environmental systems (forest, river, crop), monitoring plays a key-issue in order to cope with extreme events (forest fires, flood, etc.). In such cases, the sampling rate has to be increased in order to have a maximum of information for decision-makers. This sampling rate may also yield a full discharge of the battery. The purpose is therefore to find strategies that enable to send a maximum of information and to keep the node operationnal in the long-term. Sampling frequencies are also very specific to a given application. In addition, it is recognized and shown previously in [17] that the offered load per node helps to significantly enhance the performances (high reliability, low packet delay, low energy consumption per node) in large networks. In this paper, we assume that a wireless sensor node has a renewable energy source such as a solar panel. Note that even with this external energy supply, consumption and power management in WSN may still be problematic because of low solar radiation observed, for example, in Nordic countries.

To achieve our goal, we use one of the existing control approaches, namely the viability theory [18]. A description of the viability theory is given in section 3.4. It aims at providing the operating policies adapted to the applications contraints in terms of lifetime duration, reliability and packet delay. We have chosen the viability theory because it is conducive to the WSN management that requires: i) tradeoffs between energy, reliability and packet delay; ii) adaption to the current WSN state according to its nodes resources. For example, low energy consumption and a high reliability may require significant packet delay that does not meet the needs of low-delay applications (e.g., emergency applications). Moreover, energy produced by a “renewable” source as a solar panel may be consumed more rapidly than it is produced. So, the balance between the consumed and produced energies according to the expected performance can be achieved through the viability theory.

The application of the viability theory relies on the definition, by the manager, of an analytical model describing the dynamics of the resources or their evolution over time and of a constraint space (i.e., a set of desirable states) where the resources of a wireless sensor node have to be confined. Finally, WSN are subjected to surprises and hazards and it seems to be unrealistic to integrate an optimization algorithm that can be adapted to all possible states of the WSN: it will require to perform a new optimization at each new state of the WSN. Monitored phenomena can also require dynamic sampling frequencies (e.g., rain events in vineyard).

The outcomes of the viability theory rely on a control map, that can be aggregated, that enables the adaption of the energy consumption according to the new state of the WSN. It yields more flexibility that classical optimization algorithm and consumes less energy in terms of computations. Moreover, unlike other approaches (some related to optimizations), the viability theory recommendations could be embedded inside a wireless sensor node. Thus, remote intelligent system that provides to the nodes their operating frequencies is not necessary. So, in this paper, we discuss the analytical model describing the dynamics of the resources and then we seek to find operating policies (i.e., the controls) to be applied at the appropriate time on a wireless sensor node to preserve its viability (i.e., to maintain sustainably the dynamics of the system within its space of constraints).

In a first section, we present the related work of WSN management. We especially focus on the need of flexible and adaptive tools, one of our motivations for this study. We then present our specific WSN and the associated model, that has been previously developed. This model is then nested in the mathematical formulation of the viability problem applied to a wireless sensor node. Numerical results are analyzed and discussed in terms of controls and adaption. Conclusion and future perspectives are given in the last section.

## 2 From optimal to adaptive and viable management of WSN

In the literature, many papers targeted to assess and to improve the performance of WSN. Many schemes have been proposed to meet this issue. Most contributions, in this context, have focused on operating systems [3, 4], network layer [5–7] MAC layer [8–14] and PHY layer [15]. In this section, we give an overview of the related work on WSN management based on MAC layer.

Most reseach works [8–10] have focused on the development of MAC layers to address this issue. In [8], the authors designed a MAC protocol to guarantee and increase the reliable delivery of the high quality data (e.g., real-time data) under low energy consumption and low transmission delay. It uses a service differentiation mechanism based on a spatial correlativity model of nodes and on the perceived data by the nodes in order to select nodes close to the information and prioritize their high quality data in the access channel. The authors in [9] proposed an adaptive QoS-aware MAC protocol for WSNs with heterogeneous traffic. This protocol uses a hybrid channel access method by combining the strength of both contention-based and contention-free MAC protocols. It adjusts the behavior of its channel access method depending on network traffic loads to ensure high channel utilization and low latency. Moreover, it uses a traffic prioritization mechanism to provide efficient and fair delivery of both real-time and best-effort traffic. In [10], the authors designed a network stack (PHY, MAC and Network layers) for precision agriculture to minimize energy consumption at the transceiver. The MAC layer uses a wake-up synchronization scheme between sender and receiver to reduce energy consumption. It uses a short pulse for wake-up signal. The PHY layer uses multiple power modes (drowsy and ping modes) during the wake up synchronization phase of the communication. The Network layer balances the energy depletion over the nodes in the network. Upon receiving a packet, a node has to choose, as next hop, the node with the maximum remaining energy level among neighbours closer to the sink node. Moreover, the role of the sink node is rotated among the corner nodes of the monitoring field. Note that a poorly designed layer could cause problems (e.g., failure handling, synchronization and inter-node communications) and degrade WSN performance. So, the new layer should be properly modelled and written in such a way that it is easily portable to different hardware platforms with minimal changes.

Contrary and in complement to previous ones, some works [11, 12, 14] focused on the adaptive tuning of MAC parameters, particularly those of the IEEE 802.15.4 Standard [19]. This Standard is usually developed and proposed by each WSN OS manufacturer (e.g., TinyOS [3] and Contiki OS [4]), and thus is properly managed by each of these OSs. Moreover, the tuning of IEEE 802.15.4 MAC parameters does not require any modification of the IEEE 802.15.4 Standard and can be easily implemented on different OSs and hardware platforms. The authors of [20] showed that the performance (reliability, scalability and timeliness) of the 802.15.4 MAC is very poor when the number of contending nodes is high. However, this poor performance is not due to the Carrier Sense Multiple Access with Collision Avoidance (CSMA/CA) algorithm used by the MAC protocol to regulate the channel access, but is caused by the default parameter setting suggested by the Standard [14, 17, 21]. Indeed, it is known that the IEEE 802.15.4 may have poor performances in terms of power consumption, reliability and delay, unless the MAC parameters are properly selected [17, 22]. Many strategies for tuning the IEEE 802.15.4 MAC parameters algorithm are considered in [11, 12, 14]. The authors in [11] introduce a service differentiation mechanism of packet transmission for improving the performance of the IEEE 802.15.4 slotted CSMA/CA MAC for time critical events (e.g., the alarms). The frames are categorized into high and low priority queues by adequately tuning the values of the CSMA/CA parameters: the minimum backoff exponent (*macMinBE*), the maximum backoff exponent (*aMaxBE*) and the initial value of the contention window (*CWinit*). The goal of this paper is to reduce queuing delays of high priority traffic but not of all traffic. However, energy efficiency, which is an important issue in WSN, has not been investigated. In addition, acknowledgements are not used and other CSMA/CA parameters (such as the maximum number of backoffs *macMaxCSMABackoffs* and the maximum number of retries *aMaxFrameRetries*) affecting the behavior of slotted CSMA/CA have not been considered.

The IEEE 802.15.4 MAC unreliability problem in single-hop networks is emphasized in [12] under CSMA/CA parameters and others parameters (packet generation rate, packet size, packet error rate). They showed that the set of the MAC parameters to values beyond the maximum ones allowed by the IEEE 802.15.4 Standard helps to guarantee a delivery ratio of 100% (or very close to 100%) in some application scenarios. However, such setting leads to high latency and high energy consumption for large networks (e.g., > 40 nodes) compared to the default parameter values (i.e., defined in the Standard) or to the standard parameter set (i.e., higher values compliant with the Standard). Moreover, no adaptive mechanism for tuning (optimal or not) the IEEE 802.15.4 MAC parameters has been proposed in this paper. The authors in [14] proposed a distributed and adaptive algorithm which optimally adjusts the CSMA/CA parameters of the IEEE 802.15.4 Standard called macMinBE, macMaxCSMABackoffs and macMaxFrameRetries, described more precisely in the following. This algorithm was based on an analytical model of the beacon-enabled version (i.e., the slotted CSMA/CA) of IEEE 802.15.4 Standard proposed in [13]. This model gives the approximated analytical expressions of reliability, delay and power consumption of a node within a single-hop topology. Moreover, this model estimates the channel sensing probabilities (i.e., the busy channel probabilities *α* and *β* during the first and the second clear channel assessment (CCA) and the channel access probability *τ*) based on the physical layer measurements. This reduces drastically the computational complexity caused by the system of non-linear equations formed by *α*, *β* and *τ*. The focus of their algorithm is on the IEEE 802.15.4 optimization problem of power consumption, thus extending the WSN lifetime, while guaranteeing reliability and delay constraints in the packet transmission. The solution of this optimization problem gives the optimal MAC parameters values (*macMinBE*, *macMaxCSMABackoffs* and *macMaxFrameRetries*). The checking for the optimal MAC parameters occurs at each update of channel sensing probability, i.e., at each sensing state of node (each *aUnitBackoffPeriod* = 80 bits equivalent to 0.32 ms for the update of *τ* and when a node stays in the first Clear Channel Assessment (CCA) and second CCA for the update of *α* and *β*, respectively). This high update frequency of channel estimation, especially if the traffic is large, leads to frequent checking and potentially changes of optimal MAC parameters, and therefore to an extra non-negligible energy consumption (computation and memory storage).

Most existing contributions, based on the adaptive tuning of MAC layer parameters, concentrated on maximizing the reliability and minimizing the delay of delivered packets. This is suitable for real-time applications, but not for monitoring applications, such as agricultural application requiring specific sampling frequencies at different times. So, rather than investigate the energy optimization problem as in [14], we propose in this paper to control the sustainable use of resources (i.e., energy consumption, reliability and packet delay) of a wireless sensor node by an adaptive tuning of the IEEE 802.15.4 MAC parameters:

- macMinBE, minimum value of the backoff exponent in the CSMA/CA algorithm, symbolized by *m*_0_
- macMaxCSMABackoffs, maximum number of backoffs attempts of the CSMA/CA algorithm before declaring a channel access failure, symbolized by *m*
- macMaxFrameRetries, maximum number of retries allowed after a transmission failure, symbolized by *n*

Unlike existing contributions, where the sampling frequency (*λ*) is an input, we also propose to control (*λ*) in order to meet the agriculture and environment application requirements. In agriculture, the collection frequency of observations is very important, for example, in the characterization of the different phases of a plant development (e.g., lifting, tillering, stem elongation, flowering and maturity). Since the growth of a plant is different during these phases, the monitoring of the plant requires different sampling frequencies at an unknown date and during an unknown period. Thus, there may be an increase in frequency from one phase to another. However, a higher sampling frequency of sensor nodes may cause significant energy consumption and poor QoS leading to violations of the application requirements. Hence, the sampling frequency must be a design parameter that needs to be adjusted to meet the application requirements. In addition, it is recognized and shown in [17] that the offered load per node helps to significantly enhance the performances (high reliability, low packet delay, low energy consumption per node) in large networks.

In this paper, we assume that a wireless sensor node has a renewable source such as a solar panel. Our goal is to find the required balance between the energy consumed by the operations done by the wireless sensor node and the one produced by this renewable source. This will allow using as long as possible, in the absence of hardware failure, the WSN for new applications, especially in isolated areas. To achieve our goal, we use one of the existing control approaches, namely the viability theory [18], see section 3.4. The purpose is to adapt WSN management in order to keep a suitable reliability and delay of packets transmission while ensuring to keep enough energy for long-term use.

## 3 Defining dynamics, controls and objectives of WSN management

### 3.1 Statement of the problem

Designers of WSNs are grappling with how to integrate uncertainty into their policies of energy consumption. Indeed, failure of a wireless sensor node is mainly due to hazards and surprises. In this context, designers need tools that enable flexible and adaptive management of the energy consumption, especially when WSNs are subjected to environmental changes. Therefore we propose to use an adaptive management of energy consumption, reliability and delay in the transmissions of WSN. Adaptive management aims at managing the system according to the current state of the WSN. Such adaptive management is used in environmental systems where hazards and uncertainties are important (see [23, 24] for such examples of adaptive management). However, adaptive management requires: 1) a controlled dynamical model of WSN; 2) tools for defining suitable controls according to the state and the dynamics of WSN. Both issues are described in what follows.

### 3.2 Dynamics

#### 3.2.1 Energy

Wireless Sensor Networks (WSNs) have typically little or no fixed infrastructure as compared with wired network. So, their operations require an intelligent management of their resources to meet the lifetime expected by the deployed application. Moreover, node’s energy is generally the most critical. To estimate or control the lifetime of a node, by extension, that of the WSN, it is necessary to know at a given time the remaining energy of a node. The energy evolution of a node in discrete time, defined by a time interval *dt* of 0.2, can be written as follows:

$$\begin{array}{c} E\left(t+1\right)=E\left(t\right)+E_{sol}-E_{con}\left(t,p^{WSN}\right) \end{array}$$ (1)

Where the term *E*(*t*) is the remaining energy of a node at time *t*. The energy *E*_*sol*_ is the average external energy produced by the solar panel (an expression is given in the first item of this Section 3.2.1). It is used to partially mitigate the limitation of energy of a wireless sensor node. In our study case, we assume that *E*_*sol*_ is constant and does not depend on time and a set of other parameters symbolized by *p*^*WSN*^. *E*_*con*_ is the average energy consumption of a node at time *t* with a set *p*^*WSN*^ of other parameters. The set *p*^*WSN*^ includes parameters related to different network protocol layers of a node (i.e., mainly the physical (PHY), MAC and application layers), to the environment and to device characteristics. Indeed, in most WSNs, the nodes communicate with low power and consequently the radio links are very unreliable. This can lead to losses that may be due to the unpredictable and harsh nature of communication channels, i.e., the PHY layer (bandwidth, data/bit rate, encoding, modulation, etc.). Moreover, the shadowing and fading effects of wireless channel due to environment characteristics (ambient temperature, presence of obstacles, monitoring area) and the noise floor related to radio devices (heterogeneity and location) influence the signal strength and thereafter the link reliability. The MAC layer (contention window, buffer size, etc.) affects the network performance and the energy consumption because it is responsible for establishing communications for data transfers and also for the allocation of communication resources between the contending nodes. The application layer (e.g., sampling frequency, data frame size, etc.) affects the MAC layer and allows estimating the level of network congestion and also the energy resources. An analytical model of *E*_*con*_ has been proposed in [17] and in [25] and recalled in the second item of this Section 3.2.1 for the sake of simplicity. In what follows, for the sake of clarity, we remove the parameter *t* from the equations, keeping in mind that the parameters of the WSN depend on time. Then we will define among the WSN parameters those that we can control and therefore that evolve in time.

- Energy produced by a solar panel

The performance of a solar photovoltaic (PV) system depends upon several factors namely: the characteristics (type and size) of the solar panel, solar radiation, the inclination of the PV module relative to full sun, the ambient temperature, the wind speed, etc. The average electrical energy produced by a PV panel during a time *T* is given as follows:

$$\begin{array}{c} E_{sol}=P_{sol}\times T\times \left(\frac{Number\;of\;hours\;of\;sun\;per\;year}{365\times 24}\right) \end{array}$$ (2)

Where *P*_*sol*_ is the electrical power produced by a PV panel [2]:

$$\begin{array}{c} P_{sol}=\eta \times G\times S \end{array}$$ (3)

with

$$\begin{array}{c} \eta =\eta_{0}\times \left[1-\gamma \left(T_{cell}-T_{ref}\right)\right] \end{array}$$ (4)

$$\begin{array}{c} \eta_{0}=\frac{P_{max}}{P_{in}\times S}=\frac{V_{max}\times I_{max}}{P_{in}\times S}=\frac{FF\times V_{OC}\times I_{SC}}{P_{in}\times S} \end{array}$$ (5)

$$\begin{array}{c} G=G_{sol}\times \cos\left(\alpha \right) \end{array}$$ (6)

where *η* is the instantaneous efficiency of the PV module; *η*_0_ is the PV module efficiency at reference temperature and *γ* is the temperature coefficient for photovoltaic conversion efficiency (in %/°C); the values of *η*_0_ and *γ* are obtained from the PV characteristic curves given in the specification sheet of the manufacturer; *P*_*max*_ is the typical peak power produced by the PV module at Standard Test Conditions (STC), i.e., a cell temperature of 25°C, a illumination of 1000 W/m^2^ at spectral distribution of an Air Mass (AM) 1.5. *I*_*max*_ and *V*_*max*_ are the current and the voltage corresponding to the peak power *P*_*max*_ of the PV module; *V*_*OC*_ is the open-circuit voltage and *I*_*SC*_ is the short-circuit current; *FF* is the fill factor which gives an idea of the quality of the PV module. *P*_*in*_ is the incident power of solar radiation which is on average equal to 1000 W/m^2^; *S* is the active surface area (in m^2^) of the PV module, i.e., the total surface area of the PV cells receiving solar radiation; *T*_*cell*_ is the PV cell temperature; *T*_*ref*_ is the reference temperature (25°C); *G* is the effective solar irradiance (in W/m^2^) on the PV cell which does not correspond to the reference solar irradiance *G*_*sol*_ coming directly from the sun; the effective irradiance *G* decreases according to the sun position of the factor cos(*α*) where *α* is the angle of incidence;

The PV cell temperature *T*_*cell*_ varies depending on the irradiance *G* and the ambient temperature *T*_*a*_ of 19°C according to the relationship [26, 27]:

$$\begin{array}{c} T_{cell}-T_{a}=\left(\frac{T_{fn}-20}{800}\right)\times G \end{array}$$ (7)

where *T*_*fn*_ (in °C) is the nominal operating cell temperature (NOCT) for a solar irradiation of 800 W/m^2^, an ambient temperature of 20°C, and a wind speed of 1 m/s. The value of *T*_*fn*_ is given by the manufacturer. The parameters values of the PV module are presented in Table 1.

**Table 1:** Parameters of the PV module.

Variable	Definition	Value
*S*	Active surface of the PV module	0.075*m* × 0.24*m*
*P*_*in*_	Average incident power of solar radiation	1000*W*/*m*^2^
*V*_*max*_	Voltage corresponding to the peak power of the PV module	7.2V
*I*_*max*_	Current corresponding to the peak power of the PV module	0.10A
*γ*	Temperature coefficient for photovoltaic conversion efficiency	−0.43%/°C).
*T*_*ref*_	Reference temperature	25°C
*T*_*fn*_	Nominal operating cell temperature (NOCT) for a solar irradiation of 800 W/m^2^, an ambient temperature of 20°C, and a wind speed of 1 m/s	46°C
*T*_*a*_	Ambient temperature	19°C
*G*_*sol*_	Reference solar irradiance coming directly from the sun	249*W*/*m*^2^
*α*	Angle of incidence of PV panel	45°C
	Number of hours of sun per year	1898

- Energy consumption of a wireless sensor node

The energy consumption model is described in detail previously in [17] [25]. This model is obtained through a Markov Chain modeling the slotted binary exponential backoff process of the IEEE 802.15.4 proposed in [13]. This model takes into account energy dissipation of the radio unit and also of the processing and the sensor units in order to capture more faithfully the lifetime of a wireless sensor node. The total energy consumption of a node represented by the variable *E*_*con*_ is the energy consumed by the basic units of a node i.e., the sensing, the processing and the radio units denoted respectively by the variables: *E*_*sensor*_*unit*_, *E*_*μc*_*unit*_, *E*_*RF*_*unit*_. Then, the total energy consumption of a node, as described in detail previously in [17] [25], is given as follows:

$$\begin{array}{c} E_{con}=E_{sensor\_unit}+E_{RF\_unit}+E_{\mu c\_unit} \end{array}$$ (8)

The energy consumption of the transmission unit *E*_*RF*_*unit*_ is expressed as follows:

$$\begin{array}{ll} E_{RF\_unit}= & \frac{P_{i}\cdot \tau }{2}\cdot \left[\frac{\left(1-x\right)\left(1-{\left(2x\right)}^{m+1}\right)}{\left(1-2x\right)\left(1-x^{m+1}\right)}\cdot 2^{m_{0}}-1\right]S_{b}+P_{sc}(2-\alpha )\tau \times S_{b}\\ & +(1-\alpha )(1-\beta )\tau \times \left[P_{t}L+P_{i}+L_{ack}\cdot \left(P_{r}\cdot (1-P_{fail})+P_{i}P_{fail}\right)\right]S_{b}\\ & +P_{sp}\frac{1-p_{0}}{p_{0}}L_{0}\left[\begin{array}{l} x^{m+1}(1+y)+\\ \left(P_{fail}y^{n}+(1-P_{fail})(1+y\right))(1-x^{2}) \end{array}\right]b_{0,0,0}\cdot S_{b}\\ & +P_{sp}L_{1}\left[\begin{array}{l} x^{m+1}\cdot (1+y)+\\ \left(\begin{array}{l} P_{fail}y^{n}+\\ \left(1-P_{fail})(1+y\right) \end{array}\right)(1-x^{2}) \end{array}\right]b_{0,0,0}\cdot S_{b}\\ & +P_{w}p_{0}\left[\begin{array}{l} x^{m+1}\cdot (1+y)+\\ \left(\begin{array}{l} P_{fail}\cdot y^{n}+\\ \left(1-P_{fail})\cdot (1+y\right) \end{array}\right)(1-x^{2}) \end{array}\right]b_{0,0,0}\cdot S_{b} \end{array}$$ (9)

$$\begin{array}{c} x=\alpha +(1-\alpha )\beta \end{array}$$ (10)

$$\begin{array}{c} y=P_{fail}\left(1-x^{m+1}\right) \end{array}$$ (11)

$$\begin{array}{c} P_{fail}=1-\left(1-P_{e}\right)\left(1-P_{col}\right) \end{array}$$ (12)

$$\begin{array}{c} P_{col}=1-{\left(1-\left(1-p_{0}\right)\tau \right)}^{N-1} \end{array}$$ (13)

Where *P*_*i*_, *P*_*sc*_, *P*_*t*_, *P*_*r*_, *P*_*w*_ and *P*_*sp*_ are the power consumption of the RF unit in idle-listen state, channel sensing state, transmitting state, receiving state, wake-up state and sleep states during the backoff stages, respectively. *x* is the busy channel probability; *y* is the probability of unsuccessful packet transmission; more specifically, *y* is the probability that a packet, after successfully accessing the channel, is lost (e.g., collisions); *m*_0_ is the minimum value of backoff exponent; *m* is the maximum number of backoff stages; *n* is the maximum number of retries; *L*_0_ is the length of the idle-queue state without generating packets; *L*_1_ (an expression is proposed in [28]) is the packet copying delay between the microcontroller and the radio transceiver; *α* and *β* are the busy channel probabilities during the first and the second Clear Channel Assessment (CCA) and *τ* is the channel access probability (Eqs (1) to (3) in [29]); *p*_0_ is the steady state probability that the queue is empty (an estimation is proposed in [29]); *L* is the length of the data frame in unit backoff periods and *L*_*ack*_ is the length of ACK frame; *N* is the number of neighbouring stations; *b*_0,0,0_ is the state where the state variables of the backoff stage counter, the backoff counter and the retransmission counter are equal to 0 (an approximation is proposed in [13]); *P*_*fail*_ is the transmission failure probability; *P*_*col*_ is the collision probability and *P*_*e*_ is the transmission error probability due to PHY layer constraints; *S*_*b*_ is the time unit.

The energy *E*_*sensor*_*unit*_ dissipated by a node upon activation of its sensing unit during time unit *S*_*b*_ can be defined as follows:

$$\begin{array}{c} \begin{array}{ccc} E_{sensor\_unit} & = & \lambda S_{b}\times \sum_{i=1}^{N_{sensing}}\left(P_{on\_sensing}\left(i\right)\times t_{sensing}\left(i\right)\right)\\ & & +S_{b}\times \sum_{i=1}^{N_{sensing}}\left(P_{off\_sensing}\left(i\right)\times \left(1-\lambda t_{sensing}\left(i\right)\right)\right)\\ & & +P_{on\_ADC}\times \lambda S_{b}t_{measure}+P_{off\_ADC}\times S_{b}\left(1-\lambda t_{measure}\right) \end{array} \end{array}$$ (14)

Where *P*_*on*_*sensing*_(*i*) and *P*_*off*_*sensing*_(*i*) are respectively the power consumption related to the sensor *i* when it is active or not; *P*_*on*_*ADC*_ and *P*_*off*_*ADC*_ are respectively the ADC power consumption when it is active or not; $t_{measure}=\sum_{i=1}^{N_{sensing}}t_{sensing}\left(i\right)$ is the total duration of the measurement phase of the sensor board; *N*_*sensing*_ is the number of active sensors on the board; *t*_*sensing*_(*i*) is the time response of the physical measure associated with the active sensor *i*; *λ* denotes the sensing frequency of sensor node.

The energy consumption of the processing unit *E*_*μc*_*unit*_, i.e., the energy dissipated by a node upon activation of its data processing unit can be expressed as follows:

$$\begin{array}{ll} E_{\mu c\_unit}= & P_{on\_\mu c}\times \left[\begin{array}{l} \lambda S_{b}t_{measure}+\frac{N_{cycle}}{F_{\mu C}}S_{b}\\ +(1-\alpha )(1-\beta )(1-p_{0})\tau LS_{b}\\ +(1-\alpha )(1-\beta )(1-p_{0})(1-P_{fail})L_{ack}S_{b} \end{array}\right]\\ & +P_{off\_\mu c}\times \left[S_{b}-\left(\begin{array}{l} \lambda S_{b}t_{measure}+\frac{N_{cycle}}{F_{\mu C}}S_{b}\\ +(1-\alpha )(1-\beta )(1-p_{0})\tau LS_{b}\\ +(1-\alpha )(1-\beta )(1-p_{0})(1-P_{fail})L_{ack}S_{b} \end{array}\right)\right] \end{array}$$ (15)

Where *P*_*on*_*μc*_ and *P*_*off*_*μc*_ are the power consumption when it is respectively active or idle, respectively; the term $\frac{N_{cycle}}{F_{\mu C}}S_{b}$ corresponds to data processing stage (i.e., collection and manipulation of data) over 802.15.4 Backoff time *S*_*b*_; *N*_*cycle*_ is the average number of cycles per second according to the microcontroller speed *F*_*μC*_ and the software running on the wireless sensor node; the term *λS*_*b*_ *t*_*measure*_ corresponds to the collection of physical measurements performed by sensing units.

#### 3.2.2 Reliability and delay

The maximization of the network lifetime could significantly degrade other network performance (e.g., the communication reliability of a node, the packet delay, etc.) expected by WSN applications. Hence, it is necessary to find a trade-off between the requirements of applications and also the inherent constraints of wireless sensor nodes. Indeed, the bandwidth and memory limitations can cause large latencies and high packet loss, and therefore a low level of Quality of Service (QoS) if the network density is high. So, we propose to include in the dynamical system of a node, the average reliability (i.e., the successful frame delivery ratio) of a node and the average packet delay represented by the states *R* and *D*, respectively. The dynamic equations representing the reliability (i.e., the probability of a good frame reception) and the packet delay (i.e., the average waiting time to receive a frame) of a node have been proposed in [29], described in detail previously in [25], and given as follows:

$$\begin{array}{c} R=\left(1-p_{k}\right)\left(1-P_{cf}\right)\left(1-P_{cr}\right) \end{array}$$ (16)

$$\begin{array}{c} D=\frac{L}{\lambda \left(1-p_{k}\right)} \end{array}$$ (17)

with

$$\begin{array}{c} P_{cf}=\frac{x^{m+1}\left(1-y^{n+1}\right)}{1-y}\text{and }P_{cr}=y^{n+1} \end{array}$$ (18)

Where *p*_*k*_ is the probability of having full buffer (Eq 6 in [29]), *P*_*cf*_ is the probability that the packet is discarded due to channel failure and *P*_*cr*_ is the probability that the packet is discarded due to retry limits, *L* is the payload size, *x* is the busy channel probability (see Eq 10) and *y* is the probability of an unsuccessful packet transmission (see Eq 11); more specifically, *y* is the probability that a packet, after successfully accessing the channel, is lost (e.g., collisions); *m* is the maximum number of backoff stages and *n* is the maximum number of retries in IEEE 802.15.4 slotted CSMA/CA MAC; *λ* denotes the sensing frequency of a sensor node.

### 3.3 Controls

To control the use of the wireless sensor node resources, a number of recommendations or action policies through controllers must be applied to the node to maintain the dynamic of its system in a constraint set. However, it is important to first identify the controllers, which affect both the dynamics of a node described in Eqs 22, 23 and 24, i.e., the available energy (lifetime), the reliability and the packet delay of a wireless sensor node. This study was conducted in detail in [17] under MAC parameters (*m*_0_, *m*, *n*) where *m*_0_ denotes the minimum value of the backoff exponent in the Carrier Sense Multiple Access with Collision Avoidance (CSMA/CA) algorithm, *m* represents the maximum number of backoffs that this same algorithm will attempt before declaring a channel access failure and *n* denotes the maximum number of retries allowed after a transmission failure. The study of [17] was focused on the parameters of the MAC and the application layers. Indeed, the MAC layer helps to achieve good network performance and energy saving given that it is responsible for establishing communication for data transfers and also for the allocation of communication resources between the contending nodes. For example, it is known that IEEE 802.15.4 may have poor performances in terms of power consumption, reliability and delay [22], unless the MAC parameters are properly selected. Moreover, the application layer affects the MAC layer and allows estimating the level of network congestion and also the energy resources. The authors in [17] have shown that especially in small networks, the MAC parameters (*m*_0_, *m*) are the main keys for reliability, packet delay and energy consumption of a node in the beacon-enabled slotted CSMA/CA of IEEE 802.15.4 Standard. However, in large networks, the setting of MAC parameters (*m*_0_, *m*) do not allow to ensure a good average reliability with a low average packet delay and lower average consumption. The offered load per node could be properly tuned through, for example, the sampling frequency (symbolized by the variable *λ*) in order to obtain such performances. So, *m*_0_, *m* and *u*_*λ*_ are the controllers chosen to preserve the viability of a node with *u*_*λ*_ controlling the time variation of the sampling frequency. Indeed, we suppose that the node can act on its sampling frequency according to its remaining energy level in order to see the effects of the sampling frequency on the lifetime of node. We have therefore the following three controls that may evolve in time:

$$\begin{array}{cc} m_{0}\left(t\right) & \in [m_{0,min};m_{0,max}] \end{array}$$ (19)

$$\begin{array}{cc} m(t) & \in [m_{min};m_{max}] \end{array}$$ (20)

$$\begin{array}{cc} u_{\lambda }\left(t\right) & \in [-VL_{\lambda };VL_{\lambda }] \end{array}$$ (21)

where *V* and *L*_*λ*_ are the maximum velocity and the length of the growth rate of inputs of the sampling frequency, respectively. Note that the parameter *V* is used to control the window of *u*_*λ*_. A wider window (i.e., a wider *V*) will increase the system reactivity. The consideration of these controls in the Eqs (1, 16 and 17) gives new dynamics:

$$\begin{array}{ccc} E(t+1) & = & E\left(t\right)+E_{sol}-E_{con}\left(\lambda \left(t\right),m_{0}\left(t\right),m\left(t\right),p^{WSN}\right) \end{array}$$ (22)

$$R\left(t+1\right)=\left[\begin{array}{l} \left(1-p_{k}\left(\lambda \left(t\right),m_{0}\left(t\right),m\left(t\right),p^{WSN}\right)\right)\\ \times \left(1-P_{cf}\left(\lambda \left(t\right),m_{0}\left(t\right),m\left(t\right),p^{WSN}\right)\right)\\ \times \left(1-P_{cr}\left(\lambda \left(t\right),m_{0}\left(t\right),m\left(t\right),p^{WSN}\right)\right) \end{array}\right]$$ (23)

$$\begin{array}{ccc} D(t+1) & = & \frac{L}{\lambda \left(t\right)\left(1-p_{k}\left(\lambda \left(t\right),m_{0}\left(t\right),m\left(t\right),p^{WSN}\right)\right)} \end{array}$$ (24)

$$\begin{array}{ccc} \lambda (t+1) & = & \lambda \left(t\right)+u_{\lambda } \end{array}$$ (25)

### 3.4 Using the viability theory for managing WSN systems

#### 3.4.1 Defining adaptive management of WSN

In a WSN environment subject to uncertainties (wireless communication and hardware failures), Designers need tools that enable flexible and adaptive management of the energy consumption, especially when WSNs are subjected to environmental changes. Rather than managing for a single, optimal state, users have to manage their wireless sensor node within a range of acceptable outcomes while avoiding failure and keeping flexibility in their policy of energy consumption. Users should therefore gain by using tools that enable them to find not the optimal policy but to find a policy that avoid energy failure. We argue that viability theory [18] is a suitable tool for managing the wireless sensor node’s energy in order to avoid its energetic failures, especially when the WSN is subjected to unexpected environmental changes (fires, flood, etc.). These environmental changes can have an impact in the energy production of the renewable sources and require higher acquisition frequencies. Indeed, agricultural and environmental phenomena can generate events that need more data acquisition to well-understand them.

The viability theory aims to preserve some qualities of a dynamical system that evolves in a set of desirable states. These desirable states have to be defined beforehand in order to avoid the wireless sensor node failure. In our case, the set of the desirable states that avoid the failure is called “viability kernel” [18, 30, 31]. This theory has been successfully used for tackling environmental management issues such as forestry and fishery management [23, 31] or for managing bird community in farmland [32] or for climate change [33]. This theory handles two issues:

1. Defining the desirable states of the use of a wireless sensor node
2. Considering all the energy policies instead of one optimal policy

In this article, we seek to focus on defining “viable” energy management while keeping the following desirable states that is a minimum reliability of node communication and a maximum packet transmission delay. These desirables states are mathematically symbolized by *K*. In the viability framework, an important innovation is to introduce controls to explicitly account for the possibility to act on the system. In this framework, controls are not fixed beforehand. Instead, the goal is to find relevant management strategies (not necessarily “optimal” according to a certain criterion) that will maintain indefinitely the system into its state constraints *K*. In our case, we suppose that we can change the values of the sampling frequency and the setting of some MAC parameters (*m* and *m*_0_). In discrete time, this means that, at each time step, there is a set of possible controls that one must choose from. One objective of the viability theory is to determine which are the set of the desirable states of our WSN, denoted *K*, for which the dynamics can keep the WSN properties. This objective to keep relevant system properties is achieved through management strategies i.e., by choosing suitable values of *m*(*t*), *m*_0_(*t*) and *u*_*λ*_(*t*) at each time step. The set of all the states for which there is a control strategy such that the system can be maintained at all times inside the set of desirable states is called the viability kernel *Viab*(*K*). The viability kernel *Viab*(*K*) is composed of all initial states of the system for which there is at least a sequence of controls (*m*(*t*), *m*_0_(*t*), *u*_*λ*_(*t*)) which influences the evolution of the WSN at time *t* and allows the system to stay in this same viability kernel. In discrete time, it can be formally defined as the set of initial states for which there exists a trajectory that does not leave *K*:

$$\begin{array}{c} \begin{array}{ccc} Viab(K) & = & \left\{E(0),R(0),D(0),\lambda (0)\in K|\forall t\geq 0,\right.\\ & & \exists \left(m\left(t\right),m_{0}\left(t\right),u_{\lambda }\left(t\right)\right)\in \left[m_{0,min};m_{0,max}\right]\times \left[m_{min};m_{max}\right]\times \left[-VL_{\lambda };VL_{\lambda }\right],\\ & & \left.E(t),R(t),D(t),\lambda (t)\in K\right\} \end{array} \end{array}$$ (26)

Within the viability kernel, the system is maintained in a desirable state for so long as it is not disturbed. The computation of a viability kernel also yields the set of controls which maintain the system viable, which are called the viable controls. Thus, it incorporates the impacts of management policies, and implicitly optimizes them.

**Fig 1:**
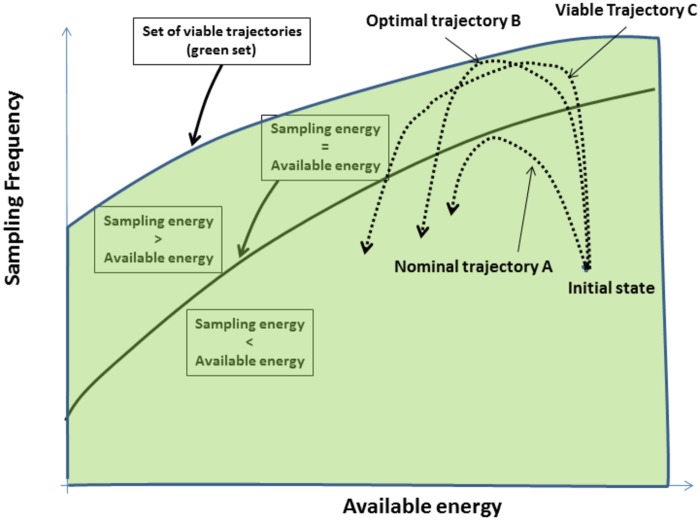
Illustration of the viability theory.

The purpose of this work is to study alternative solutions to optimization-based solutions. Indeed, using “classical” optimisation methods, at every node state change, requires more resources that a wireless sensor has. For some problems, even a single computer is not sufficient to obtain a result in acceptable time. The idea is therefore not to optimize the system but to find all strategies that enables to comply with the constraints. Theoretically, the viability set could, in turn, be integrated in a wireless sensor: it is possible to increase the sampling rate until this viability boundary. As shown in Fig 1, from the initial state, a wireless sensor node can adapt its sampling frequency to follow “Nominal trajectory A”. This trajectory belongs to the part (under the green line) of the viability kernel (the green area under the blue line) where the energy consumed for sampling is less than the available energy. However, the node can “switch” to the “Viable Trajectory C” with higher sampling frequencies in order to better follow the changes in the monitored phenomenon (e.g., a flood on a watershed). Even if the sampling energy is higher than the available one, staying in the viability kernel implies that, at each step, there exists a set of controls that will maintain the node in a viable state. As we can see, the “Optimal trajectory B” also belongs to the viability kernel.

#### 3.4.2 Defining the desirable states and the control

The objective is to maintain in a desired state the evolution of the system described by Eqs 22 to 25 and to define mathematically the constraint set *K*. We assume that the node is working when its remaining energy exceeds a given threshold *E*_*min*_, i.e., the positive variable *E* satisfies:

$$\begin{array}{c} E\in \left[E_{min};E_{batt}\right] \end{array}$$ (27)

We assume that the objective of the communication reliability of a node is reached when the positive variable *R* is higher than a minimum reliability *R*_*min*_:

$$\begin{array}{c} R\in \left[R_{min};1\right] \end{array}$$ (28)

We also assume that the objective of the packet transmission delay is reached when the positive variable *D* is below the acceptable delay *D*_*max*_:

$$\begin{array}{c} D\in ]0;D_{max}] \end{array}$$ (29)

We assume that the sampling frequency *λ* of node is a positive variable, which satisfies:

$$\begin{array}{c} \lambda \in \left[\lambda_{min};\lambda_{max}\right] \end{array}$$ (30)

## 4 Results and discussions

In this section, we study the viability of the dynamical system (described by Eqs 22 to 25) of a node into a star (i.e., single-hop) topology WSN (composed of *N* wireless sensor nodes) by using the viability algorithm proposed in [34]. The node investigated here is a Libelium Waspmote microsensor [35] rather than another (as Micaz [36], etc.) because of two reasons. Firstly, the Libelium Waspmote device encompasses sensor boards such as the agriculture board, which allows monitoring multiple environmental parameters (e.g., air temperature and humidity, soil moisture, leaf wetness and atmospheric pressure, etc.) for agricultural and environmental applications. Secondly, the Libelium Waspmote device has high power consumption. So, it is important to adopt a dynamical energy management policy with the objective of extending its durability after the deployment.

Note that the analytical models *E*_*con*_, *R* and *D* (see Eqs 8, 16 and 17) which are used to analyze the dynamics (Eqs 22 to 25) are those of a wireless sensor node into a star topology (i.e., single-hop) network under the beacon-enabled slotted CSMA/CA of IEEE 802.15.4 Standard in non-saturation mode with acknowledged uplink (communications are from wireless sensor nodes to a personal area network coordinator) and limited buffers. These analytical models provide a more precise computation of the average communication reliability, the average delay and the average energy consumption of a node because they take into account parameters, such as those related to: the network density, the offered load per node, the medium access control (MAC), the effects of PHY layer (the shadowing and fading effects of wireless channel due to environment characteristics, the noise floor related to radio devices, the modulation and encoding mechanisms used by the radio to send data over the wireless channel, etc.), etc.

Tables 2–4 presents the parameters of the model described by Eqs 22 to 25. The following sections present the results of the viability kernel of the set *K* of state constraints denoted *Viab*(*K*) and of the trajectories from *K* into *Viab*(*K*) governed by a set of admissible controls. Results are presented for a node into a star topology network of 100 nodes. We have achieved an extensive set of simulations in which we do vary several variables such as:

- the energy produced by a solar panel during a time unit *S*_*b*_ (i.e., the backoff time unit in beacon-enabled slotted CSMA/CA of IEEE 802.15.4 Standard, *S*_*b*_ = 0.32 ms): *E*_*sol*_ = 2 *μ*J/slot to 10 *μ*J/slot;
- the minimum reliability of a node: *R*_*min*_ = {0.9; 0.95; 0.99};
- the maximum packet delay: *D*_*min*_ = {0.02; 0.05; 0.07} seconds.

**Table 2:** Set of state constraint *K* (see Eqs 22 to 25).

Variable	Definition	Value
*E*_*max*_	Maximum viability constraint on the remaining energy of a node	Maximum energy of a battery: *E*_*battery*_ = 30636 J
*E*_*min*_	Minimum viability constraint on the remaining energy of a node	20% of *E*_*max*_ = 6127.2 J
*R*_*min*_	Minimum viability constraint on the reliability of a node	{0.9; 0.95; 0.99}
*D*_*max*_	Maximum viability constraint on the packet delivery delay	{0.02; 0.05; 0.07} s
*λ*_*min*_	Minimum viability constraint on the sampling frequency of a node	0.1 packets/s
*λ*_*max*_	Maximum viability constraint on the sampling frequency of a node	0.5 packets/s

**Table 3:** Set of admissible controls *U* (see Eqs 22 to 25).

Variable	Definition	Value
*m*_0,*min*_	Minimum viability control on the parameter *m*_0_ of the MAC layer	2
*m*_0,*max*_	Maximum viability control on the parameter *m*_0_ of the MAC layer	8
*m*_*min*_	Minimum viability control on the parameter *m* of the MAC layer	0
*m*_*max*_	Maximum viability control on the parameter *m* of the MAC layer	5
*V*	Maximal admissible velocity of inputs of sampling frequency	[0; 9]
*L*_*λ*_	Maximal length of the growth rate of inputs of sampling frequency	$\frac{\lambda_{max}-\lambda_{min}}{50}$

**Table 4:** Set of parameters *P* (see Eqs 22 to 25).

Variable	Definition	Value
*E*_*sol*_	Energy produced by the solar panel during a slot. A slot is defined by a time unit *S*_*b*_ = 0.32 ms	[2; 10] *μ*J/slot
*E*_*con*_	Map that computes the average energy consumption of a node	They depend on *λ*(*t*), *m*_0_, *m* and others fixed parameters (related to physical layer, network size, packet size, etc.) whose values are provided in [17]
*R*	Map that computes the average reliability of node	Same note as for *E*_*con*_
*D*	Map that computes the average packet delivery delay of node	Same note as for *E*_*con*_

### 4.1 Equilibria

Viability implies that the trajectory of the evolution *z*(.) must remain always confined in a constrained set. In some cases, evolution could converge to stable states when *t* ↦ +∞. In this case, viability is partly linked to stationarity of the system, i.e., the existence of a set of fixed viable points called equilibria where the evolution arriving at these points remains inert. Thus, in this section, we seek to identify the viable stationary points *z*_*eq*_ = (*E*_*eq*_, *R*_*eq*_, *D*_*eq*_, *λ*_*eq*_) for a later analysis of our results in the next sections:

$$\begin{array}{c} \left\{\begin{array}{c} \lim_{t\to \infty }E\left(t\right)=E_{eq}\\ \lim_{t\to \infty }R\left(t\right)=R_{eq}\\ \lim_{t\to \infty }D\left(t\right)=D_{eq}\\ \lim_{t\to \infty }\lambda \left(t\right)=\lambda_{eq} \end{array}\right. \end{array}$$ (31)

**Fig 2:**
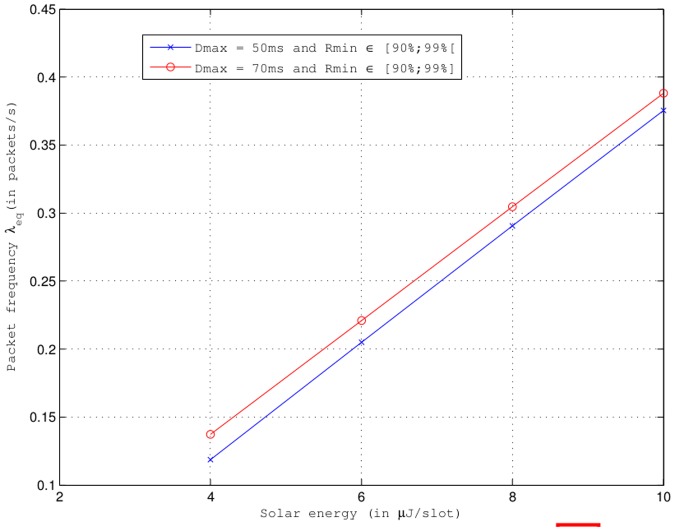
Sampling frequency input (*λ*_*eq*_) that satisfies Eq 31 vs. solar energy for different *D*_*max*_ and *R*_*min*_. No solution when: (1) *E*_*sol*_ = 2 *μ*J/slot and (2) *E*_*sol*_ = 10 *μ*J/slot, *D*_*max*_ = 50 ms and *R*_*min*_ = 99%.

Fig 2 presents the value of the sampling frequency inputs (*λ*) that satisfies Eq 31 in function of the solar energy (*E*_*sol*_) for different minimum reliability (*R*_*min*_) and maximum delay (*D*_*max*_) values. When *E*_*sol*_ = 2 *μ*J/slot, we note in Fig 2 that there is no value *λ*_*eq*_ that satisfies Eq 31. However, *λ*_*eq*_ exists and increases as *E*_*sol*_ increases (≥ 4 *μ*J/slot) and whatever *R*_*min*_ and *D*_*max*_. Indeed, the contribution of the solar panel is enough to compensate the energy dissipated in the node. In addition, the higher the value of *E*_*sol*_ is, higher will be the value of *λ*_*eq*_ for Δ*E*/Δ*t* = 0 because in communication theory and computer science, the amount of energy dissipated in the wireless sensor node depends mainly on the amount of information (during stages of sensing, processing and transmission). We can see in Fig 2 that for a given *E*_*sol*_ (≥ 4 *μ*J/slot), only *D*_*max*_ has an influence on the value of *λ*_*eq*_ because *λ*_*eq*_ varies and increases with an increase of *D*_*max*_ whatever *R*_*min*_ (≥ 90%). Indeed, when the delay constraint is less critical with a high reliability, the power consumption decreases, and subsequently the sampling frequency should be larger so that the difference between the energy produced by the solar panel and the energy dissipated in the node is null (i.e., Δ*E*/Δ*t* = 0). This explains the variation of *λ*_*eq*_ with an increasing *D*_*max*_. However, the value of *λ*_*eq*_ remains stable even with an increasing *R*_*min*_ for a given *D*_*max*_ as shown in Fig 2 because the power consumption is almost similar as *R*_*min*_ increases, and it does not require a larger value of sampling frequency for Δ*E*/Δ*t* = 0.

Table 5 shows the largest admissible sampling frequency in function of the minimum reliability *R*_*min*_, and the maximum delay *D*_*max*_ required by an application. We note in this table that the largest of *λ*-values decreases as *R*_*min*_ increases or *D*_*max*_ decreases. Indeed, the authors in [17] have shown that, in large networks, the offered load per node (i.e., the product of sampling frequency and data frame size) is a key parameter on network performance and nodal energy consumption. Moreover, the maximum offered load per node should be lower in order to have a higher reliability with lower packet delay. So, we can say that *R*_*min*_ and *D*_*max*_ have an impact on the viability kernel and also on the trajectories of viable states as they affect the sampling frequency inputs (*λ*).

**Table 5:** Largest sampling frequency inputs *λ* (in packets/s) for different values of *R*_*min*_ and *D*_*max*_.

Min reliability (*R*_*min*_)	Max Delay (*D*_*max*_)	Largest frequency (*λ*)
90%	50 ms	*λ*_*max*_ = 0.5
90%	70 ms	*λ*_*max*_ = 0.5
95%	50 ms	0.468
95%	70 ms	*λ*_*max*_ = 0.5
99%	50 ms	0.348
99%	70 ms	0.428

### 4.2 Viability kernels

This part is devoted to the experimentation per simulation of the viability kernel of a dynamical system of a node into a personal area network (PAN) for a given application scenario requiring a minimum reliability (*R*_*min*_) and a maximum packet delivery delay (*D*_*max*_). Our aim is to determine all the states from which there exists at least one trajectory satisfying Eqs 22 to 25 remaining in *K* (Eqs 27 to 30) until time *T*. To better analyze the dynamical system, we observe it in spaces in two and three dimensions.

#### 4.2.1 Viability kernel in 2-dimensional space (*E*, *λ*)

**Fig 3:**
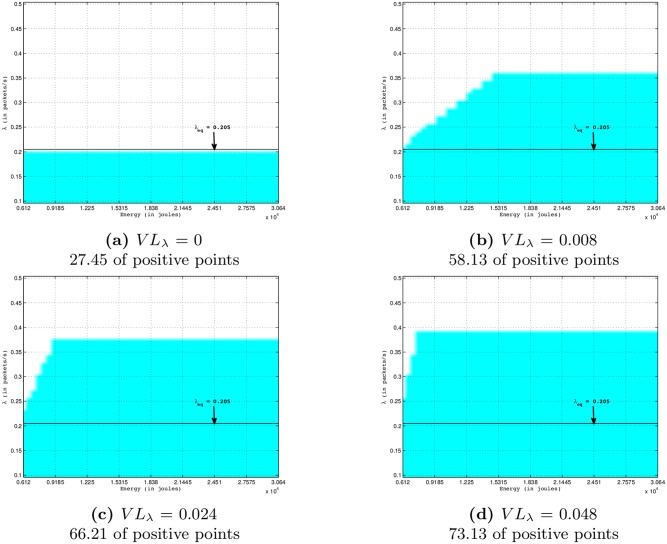
Viability kernels in 2-dimensional space (*E*, *λ*) of four-dimensional viability problem described in Eqs 22 to 25 by using values *D*_*max*_ = 50 ms, *R*_*min*_ = 0.99, *E*_*sol*_ = 6 *μ*J/slot. The kernel viability is the blue shape into the constraint space (bordered by a black rectangle). The sampling frequency equilibrium in this configuration is *λ*_*eq*_ = 0.205 packets/s (see Fig 2).

Fig 3 shows the evolution of the surface of the viability kernel (the blue shape) according to the intensity (*VL*_*λ*_) of the time variation of the sampling frequency inputs. The viability kernels on this Figure are cuttings in a two-dimensional space (*E*, *λ*), with *K* = [*E*_*min*_;*E*_*batt*_] × [*λ*_*min*_;*λ*_*max*_], of the dynamical system described in Eqs 22 to 25 by using values *R*_*min*_ = 0.99, *D*_*max*_ = 50 ms, *E*_*sol*_ = 6 *μ*J/slot. On this Figure, the x-axis and y-axis indicate the remaining nodal energy and the sampling frequency, respectively. We can see that the percentage of the kernel viability increases with *VL*_*λ*_. Indeed, an increase of *VL*_*λ*_ allows a greater decrease of the sampling frequency inputs and of the energy consumption of a node, and therefore an increase of the remaining nodal energy. So, more *VL*_*λ*_ is large, higher will be the number of viable points whose the initial remaining node energy and the initial sampling frequency tend towards *E*_*min*_ and *λ*_*max*_, respectively. This explains an increase of the surface of the viability kernel as *VL*_*λ*_ increases. Thus, as shown in Fig 3, the increase of viability kernel will enable the node to send packets with a sampling frequency larger than the sampling frequency equilibrium *λ*_*eq*_ (0.205 packets/s in this configuration; Note that 0.205 is the equilibrium value of the sampling frequency (*λ*) that satisfies Eq 31 for *R*_*min*_ = 0.99, *D*_*max*_ = 50 ms and *E*_*sol*_ = 6 *μ*J/slot as shown in Fig 2) without violating application constraints in terms of lifetime, reliability and packet delay.

#### 4.2.2 Viability kernel in 3-dimensional space (*E*, *D*, *λ*)

**Fig 4:**
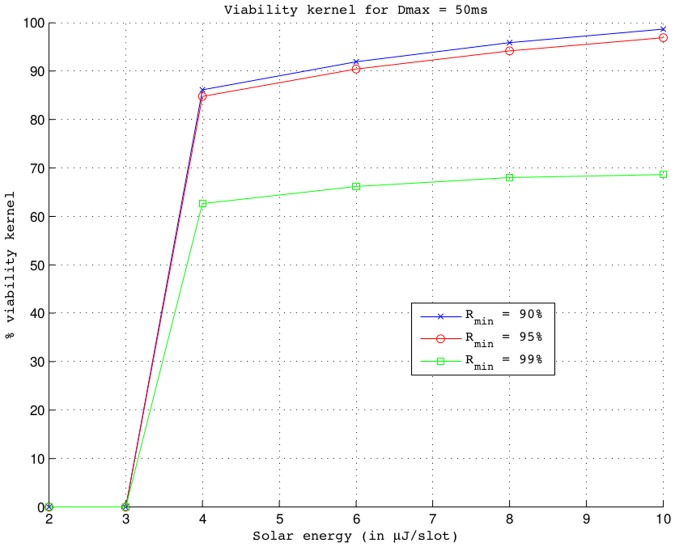
Percentage of the viability kernel in the 3-dimensional space (*E*, *D*, *λ*) vs. solar energy for *D*_*max*_ = 50 ms, *VL*_*λ*_ = 0.024 and different values of *R*_*min*_.

Fig 4 presents the percentage of the viability kernel under different values of parameters (*E*_*sol*_, *R*_*min*_). *R*_*min*_ = {0.9; 0.95; 0.99} takes three values corresponding to different application requirements and *E*_*sol*_ takes five different values in the interval [2; 10] *μ*J/slot corresponding to five solar panels with different sizes. The viability kernels percentage presented on this Fig 4 are cuttings in the three-dimensional space (*E*, *D*, *λ*) from *R*_*min*_ of a kernel viability of four-dimensional viability problem described in Eqs 22 to 25. The maximal intensity of the time variation of the sampling frequency inputs is *VL*_*λ*_ = 0.024. The maximum delay *D*_*max*_ is set to 50 ms. The volume of these viability kernels is presented in Fig A in S1 File, where the x-axis, y-axis and z-axis indicate the remaining nodal energy, the packet delivery delay and the sampling frequency, respectively. We can see in Fig 4 that the viability kernel is empty when *E*_*sol*_ ≤ 3 *μ*J/slot because the contribution of the solar panel is not enough to compensate the energy dissipated in the node (i.e., Δ*E*/Δ*t* < 0). However, when *E*_*sol*_ ≥ 4 *μ*J/slot, the viability kernel is a non-empty subset of the set of state constraints (i.e., 0 < *Viab*(*K*) < *K*). Indeed, for *E*_*sol*_ ≥ 4 *μ*J/slot, there is at least one of *λ*-values of the constraint space which satisfy the inequality Δ*E*/Δ*t* > 0 because the sampling frequency equilibrium (*λ*_*eq*_) exists (see Fig 2). Moreover, when *E*_*sol*_ increases, larger will be the *λ*-values associated with the lowest *E*-values which satisfy the inequality Δ*E*/Δ*t* > 0 because *λ*_*eq*_ increases with *E*_*sol*_ as shown in Fig 2. It is for this reason that the viability kernel increases with an increasing of *E*_*sol*_ whatever *R*_*min*_ as shown in Fig 4. We also notice that the viability kernel degrades as the required minimum reliability *R*_*min*_ increases and *E*_*sol*_ ≥ 4 *μ*J/slot. Higher the value of *R*_*min*_ is, lower will be the sampling frequency to ensure this minimum reliability and a maximum packet delivery delay *D*_*max*_ (see Table 5) and thus lower will be the number of *λ*-values meeting these requirements (see Fig A in S1 File). This explains the decrease of the viability kernel with an increase of *R*_*min*_ as shown in Fig 4.

#### 4.2.3 Viability kernel in 3-dimensional space (*E*, *R*, *λ*)

**Fig 5:**
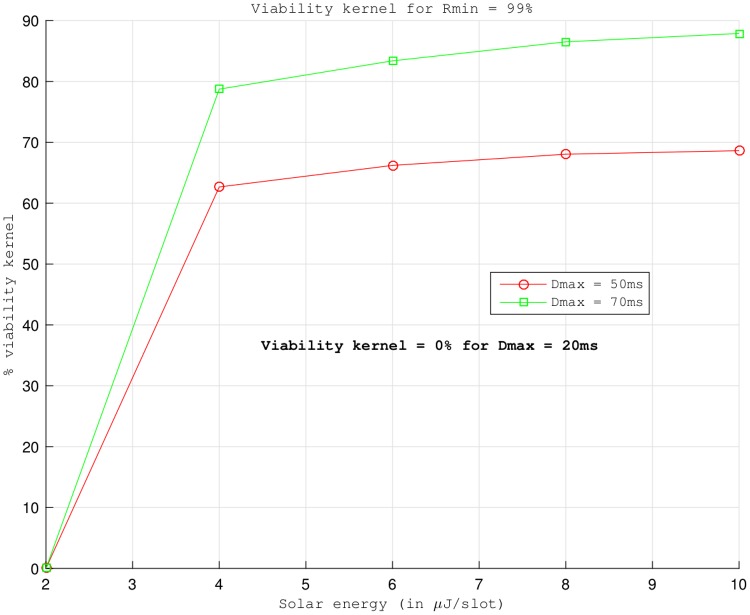
Percentage of the viability kernel in the 3-dimensional space (*E*, *R*, *λ*) vs. solar energy for *R*_*min*_ = 99%, *VL*_*λ*_ = 0.024 and different values of *D*_*max*_.

Fig 5 presents the evolution of the viability kernel percentage under different parameters (*E*_*sol*_, *D*_*max*_). *D*_*max*_ takes three values (20, 50 and 70 ms) corresponding to different application requirements and *E*_*sol*_ takes five different values in the interval [2; 10] *μ*J/slot corresponding to five solar panels with different sizes. The viability kernels presented on this Fig 5 are cuttings in the three-dimensional space (*E*, *R*, *λ*) from *D*_*max*_ of a kernel viability of four-dimensional viability problem described in Eqs 22 to 25. The maximal intensity of the time variation of the sampling frequency inputs is *VL*_*λ*_ = 0.024. The minimum reliability *R*_*min*_ is set to 99%. The volume of these viability kernels is presented in Fig B in S1 File, where the x-axis, y-axis and z-axis indicate the remaining nodal energy, the communication reliability of the node and the sampling frequency, respectively. We can see in this Fig 5 that the viability kernel is empty when *D*_*max*_ = 20 ms even with an increase of *E*_*sol*_. This is due to the chosen value of *λ*_*min*_. For a star topology WSN of 100 nodes, *λ*_*min*_ = 0.1 packets/s does not ensure both a low packet delay smaller than 20 ms and a reliability higher than 99%. This has been shown in [17] that the sampling frequency per node should be lower when the network is denser in order to have both a high reliability and a low packet delay. We also notice in Fig 5 that, for a given value of *D*_*max*_ (e.g., 50 ms and 70 ms), the viability kernel increases slowly and remains stable (its value depends on *D*_*max*_) even with an increasing of *E*_*sol*_. This is because for a given *D*_*max*_, the higher frequency sampling (represented by *λ*_*D*_) associated with *R*_*min*_ is smaller than *λ*_*max*_ (see Fig B in S1 File) and is reached when *E*_*sol*_ = 4 *μ*J/slot (see Fig 5). So, higher the solar energy is, closer to *λ*_*D*_ will be the higher sampling frequency associated to *E*_*min*_. As *λ*_*D*_ is low, the viability kernel increases slowly when *E*_*sol*_ increases. We also note in Fig 5 that the viability kernel increases as *D*_*max*_ increases. Higher the value of *D*_*max*_ is, higher can be the sampling frequency to respect this maximum packet delivery delay and a minimum reliability *R*_*min*_ (see Table 5) and thus larger will be the number of *λ*-values meeting these requirements (see Fig B in S1 File). This explains the increase of the viability kernel as *D*_*max*_ increases (see Fig 5).

### 4.3 Trajectories

**Fig 6:**
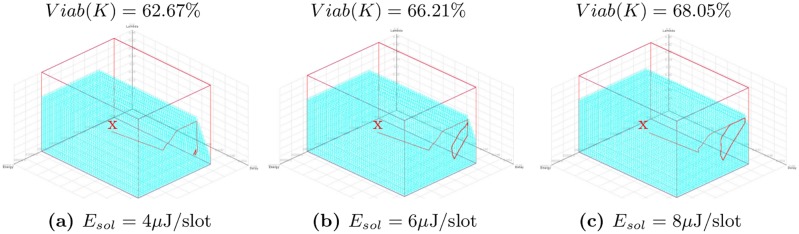
Trajectories in 3-dimensional space (*E*, *D*, *λ*) starting from a viable state *x* = (*E* = 20000 J, *D* = 20 ms, *λ* = 0.22 packets/s) controlled by Eqs 22 to 25 for *D*_*max*_ = 50 ms, *R*_*min*_ = 99%, *E*_*sol*_ = {4; 6; 8} *μ*J/slot, *VL*_*λ*_ = 0.024.

Fig 6 shows trajectories starting from a viable state *x* = (*E* = 20000 J, *D* = 20 ms, *λ* = 0.02 packets/s) using *D*_*max*_ = 50 ms, *VL*_*λ*_ = 0.024, *R*_*min*_ = 99% and *E*_*sol*_ = {4; 6; 8} *μ*J/slot where the x-axis, y-axis and z-axis indicate the remaining nodal energy, the packet delivery delay and the sampling frequency, respectively. We note that the trajectory grows up to a border, make a turn and move along the border until it reaches an area where it loops. So, the trajectory starting from initial state *x* is cyclic and no stable whatever the value of *E*_*sol*_. Indeed, the stability is obtained when *λ* = *λ*_*eq*_ (∼0.2906 packets/s for *R*_*min*_ = 99% and *D*_*max*_ = 50 ms as shown in Fig 2). However, all the *λ*-values (51 values in our study case and the length between two close values is $L_{\lambda }=\frac{\lambda_{max}-\lambda_{min}}{50}$) inside the constraint space [*λ*_*min*_; *λ*_*max*_] = [0.1; 0.5] packets/s of *λ* do not equal to *λ*_*eq*_. This explains the cyclic effect and allows sending packets to sampling frequencies larger than *λ*_*eq*_. We also observe in Fig 6 that the higher the value of *E*_*sol*_ is, larger will be the cycle because the viability kernel is larger and the number of *λ*-values which satisfy the inequality Δ*E*/Δ*t* > 0 is larger as shown Table 5.

**Fig 7:**
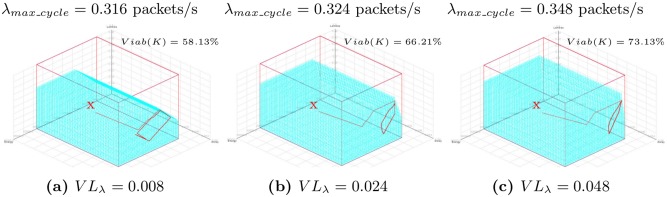
Trajectories in 3-dimensional space (*E*, *D*, *λ*) starting from a viable state *x* = (*E* = 20000 J, *D* = 20 ms, *λ* = 0.22 packets/s) controlled by Eqs 22 to 25 for *D*_*max*_ = 50 ms, *R*_*min*_ = 99%, *E*_*sol*_ = 6 *μ*J/slot, *VL*_*λ*_ = {0.008; 0.024; 0.048}.

**Fig 8:**
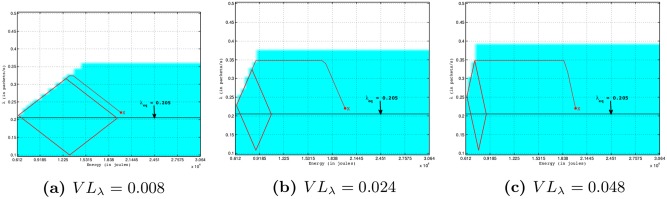
Trajectories in 2-dimensional space (*E*, *λ*) starting from a viable state *x* = (*E* = 20000 J, *λ* = 0.22 packets/s) controlled by Eqs 22 to 25 for *D*_*max*_ = 50 ms, *R*_*min*_ = 99%, *E*_*sol*_ = 6 *μ*J/slot, *VL*_*λ*_ = {0.008; 0.024; 0.048}.

Fig 7 shows for different *VL*_*λ*_, trajectories in a 3-dimensional space (*E*, *D*, *λ*) starting from a viable state *x* = (*E* = 20000 J, *D* = 20 ms, *λ* = 0.02 packets/s) using *D*_*max*_ = 50 ms, *R*_*min*_ = 99% and *E*_*sol*_ = 6 *μ*J/slot. The x-axis, y-axis and z-axis indicate the remaining nodal energy, the packet delivery delay and the sampling frequency, respectively. For the sake of clarity, the trajectories starting from the same viable state *x* are also represented in a 2-dimensional space (*E*, *λ*) as shown in Fig 8. We note that the trajectory starting from initial viable state *x* finally reaches a cyclic area. The cycle is faster when the intensity *VL*_*λ*_ is high because of larger decrease and increase of the sampling frequency inputs. Indeed, from a high sampling frequency, when the remaining nodal energy is low, a large decrease is needed in order to maintain the dynamic of the system within the viability kernel. We also observe in Fig 7 that, the maximum sampling frequency (*λ*_*max*_*cycle*_) of this cyclic area increases with an increasing *VL*_*λ*_ and is larger than *λ*_*eq*_ (0.205 packets/s in this configuration as shown in Fig 8; Note that 0.205 is the value of the sampling frequency (*λ*) that satisfies Eq 31 for *D*_*max*_ = 50 ms, *R*_*min*_ = 99% and *E*_*sol*_ = 6 *μ*J/slot as shown in Fig 2). This is due to the fact that, when *VL*_*λ*_ increases, the percentage of the kernel viability increases as explained in the section 4.2.1. This shows the importance of the viability theory because a node can send packets with a sampling frequency larger than *λ*_*eq*_ without violating application constraints in terms of lifetime, reliability and packet delay.

**Fig 9:**
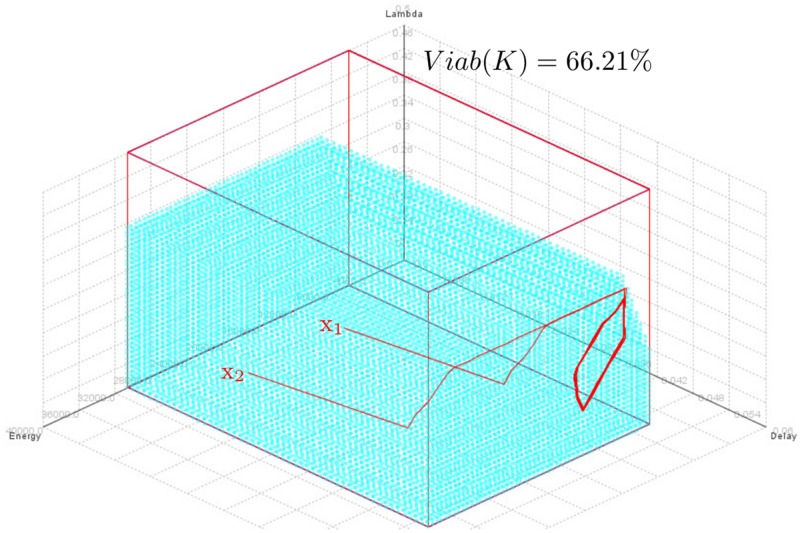
Trajectories in 3-dimensional space (*E*, *D*, *λ*) starting from two viable states *x*_1_ = (*E* = 20000 J, *D* = 20 ms, *λ* = 0.22 packets/s) and *x*_2_ = (*E* = 30636 J, *D* = 20 ms, *λ* = 0.22 packets/s) controlled by Eqs 22 to 25 for *D*_*max*_ = 50 ms, *R*_*min*_ = 99%, *E*_*sol*_ = 6 *μ*J/slot, *VL*_*λ*_ = 0.024.

**Fig 10:**
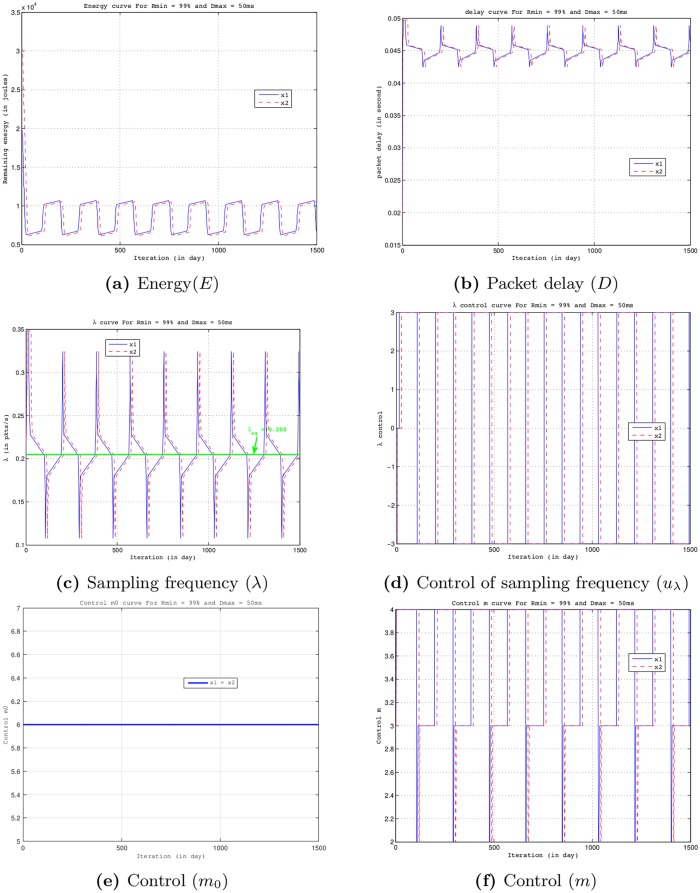
Evolution of dynamics and controls starting from two viable states *x*_1_ = (*E* = 20000 J, *D* = 20 ms, *λ* = 0.22 packets/s) and *x*_2_ = (*E* = 30636 J, *D* = 20 ms, *λ* = 0.22 packets/s) controlled by Eqs 22 to 25 for *D*_*max*_ = 50 ms, *R*_*min*_ = 99%, *E*_*sol*_ = 6 *μ*J/slot, *VL*_*λ*_ = 0.024.

Fig 9 shows trajectories starting from two viable states *x*_1_ = (*E* = 20000 J, *D* = 20 ms, *λ* = 0.02 packets/s) and *x*_2_ = (*E* = 30636 J, *D* = 20 ms, *λ* = 0.22 packets/s) using *D*_*max*_ = 50 ms, *R*_*min*_ = 99%, *VL*_*λ*_ = 0.024, *E*_*sol*_ = 6 *μ*J/slot. The x-axis, y-axis and z-axis indicate the remaining nodal energy, the packet delivery delay and the sampling frequency, respectively. We note that the initial nodal energy has no impact on the trajectory because the trajectory finally loops in the same area regardless of the initial node energy as shown in Fig 9. However, as shown in Fig 10, the initial nodal energy has an influence on the cost of a trajectory (i.e., distance between the trajectory followed by the system and the objective or the constraints imposed on it: maintaining the property before time *T*). Indeed, the higher the initial nodal energy is, later will be the next change controls to meet the objectives (see Fig 10d and 10f) and therefore lower will be the cost associated to a trajectory. We can also see in Fig 10c that a node can send packets to sampling frequencies (up to *λ*_*max*_*cycle*_ = 0.324 packets/s as shown in Fig 8b) larger than *λ*_*eq*_ (0.205 packets/s in this configuration as shown in Fig 2) while durably using its resources and preserving the dynamics of the system within the viability kernel, hence the contribution of the viability theory.

**Fig 11:**
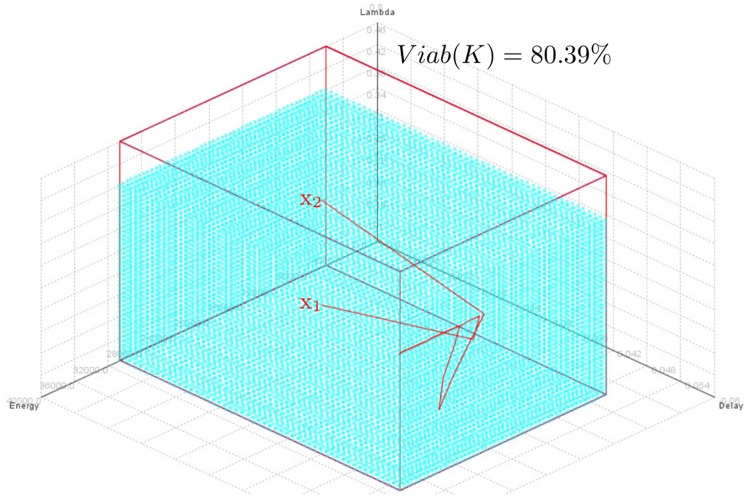
Trajectories in 3-dimensional space (*E*, *D*, *λ*) starting from two viable states *x*_1_ = (*E* = 20000 J, *D* = 20 ms, *λ* = 0.22 packets/s) and *x*_2_ = (*E* = 20000 J, *D* = 20 ms, *λ* = 0.412 packets/s) controlled by Eqs 22 to 25 for *D*_*max*_ = 50 ms, *R*_*min*_ = 99%, *E*_*sol*_ = 10 *μ*J/slot, *VL*_*λ*_ = 0.072.

Fig 11 shows trajectories starting from two viable states *x*_1_ = (*E* = 20000 J, *D* = 20 ms, *λ* = 0.22 packets/s) and *x*_2_ = (E = 20000 J, *D* = 20 ms, *λ* = 0.412 packets/s) using *D*_*max*_ = 50 ms, *R*_*min*_ = 99%, *VL*_*λ*_ = 0.072, *E*_*sol*_ = 10 *μ*J/slot and where the x-axis, y-axis and z-axis indicate the remaining nodal energy, the packet delivery delay and the sampling frequency, respectively. Note that in this configuration where *D*_*max*_ = 50 ms, *R*_*min*_ = 99% and *E*_*sol*_ = 10 *μ*J/slot, there is no equilibrium point as shown in Fig 2 because for all *λ*-values which satisfy *D*_*max*_ and *R*_*min*_, the contribution of this solar panel of 10 *μ*J/slot is enough to compensate the energy consumption of node (i.e., Δ*E*/Δ*t* > 0). In our study case, this equilibrium point *x*_*eq*_ is obtained when *D*_*eq*_ ∈ [50; 70] ms and *λ*_*eq*_ ∈ [0.35; 0.4] packets/s. So, the equilibrium point *x*_*eq*_ is outside the kernel viability and space constraints although *λ*_*eq*_ ∈ [0.35; 0.4] ⊂ [*λ*_*min*_; *λ*_*max*_] packets/s because *D*_*eq*_ ∈ [50; 70] ⊄ [0; *D*_*max*_] ms. We see in Fig 11 that the trajectories *x*_1_ (case where *λ* < *λ*_*eq*_) and *x*_2_ (case where *λ* > *λ*_*eq*_) eventually reach a same point (*E* = *E*_*max*_ = 30636 J, *D* = 49.692 ms, *λ* = 0.348 packets/s) even without an equilibrium point. Indeed, the value *λ* = 0.348 packets/s corresponds to the largest admissible sampling frequency for *R*_*min*_ = 99% and *D*_*max*_ = 50 m as shown in Table 5. Moreover, for a solar panel of 10 *μ*J/slot, Δ*E*/Δ*t* > 0 when *λ* = 0.348 packets/s. It is for this reason that, for all *λ*-values, the remaining nodal energy *E* reaches the maximum energy *E*_*max*_ of a battery (30636 J for a Lithium-ion battery of 2300mA with 3.7V nominal voltage). Thus, we can see that the initial sampling frequency of a node has no impact on the trajectory. In addition, in the absence of an equilibrium point, it has no significant influence on the cost of the trajectory because the controls remain constant.

## 5 Conclusion and perspectives

In this paper, we have investigated the performance of IEEE 802.15.4 Wireless Sensor Network (WSN). We have assumed that a wireless sensor node has a renewable energy source such as a solar panel. Despite this external energy supply, consumption and power management in WSN is still problematic because energy produced by a renewable source is generally consumed more rapidly than it is produced. So, in this paper, we have proposed an adaptive tuning of the MAC parameters (*macMinBE*, *macMaxCSMABackoffs* and *macMaxFrameRetries*) and the offered load per node in order to find the required balance between the energy consumed by the node’s operations and that produced by this renewable source, which durably guarantee the expected application requirements in terms of communication reliability and packet delay. To achieve this goal, we have used the viability theory which is a control approach. Firstly, we have proposed an analytical model describing the dynamic (evolution over time) of the energy, the communication reliability and the packet transmission delay. Then, we seek to find operating policies or controls (i.e., MAC parameters and offered load per node) to be applied at the appropriate time on a wireless sensor node in order to preserve its viability (i.e., to durably maintain its activity within a space of desired constraints).

The numerical results have shown that our solution allows ensuring indefinitely, without considering hardware failure, the operations (lifetime duration, communication reliability and packet transmission delay) of a large star topology 802.15.4 WSN composed of 100 nodes. This is beneficial for new applications deployed in isolated areas. In addition, with our viability approach, a node can send packets with a sampling frequency much higher than the equilibrium one without violating application constraints in terms of lifetime, reliability and packet delay. This is very important for real agricultural and environmental applications because wireless sensor nodes can be equipped with solar panels with better suited sizes, saving space (and even money) by a good initial choice while keeping extra energy in case of unforecasted situations. Moreover, compared to an optimization scheme as in [14], our approach allows reducing the extra energy consumption caused by the computation and the storage in memory of controls. Indeed, in our approach, controls are kept constant as long as the viability of the system is not threatened, i.e., as long as the performances of the node are inside the viability kernel. Even we specifically applied our current approach to the IEEE 802.15.4 WSN, we argue that our approach is easily transferable to other types of WSN.

As future works, we plan to include in our adaptive scheme the dynamic of the external energy supply from a solar panel since the solar irradiation varies over time. As in many wireless applications, the operations of a node could be disturbed by certain elements (e.g., the environment, the obstacles, the changes in the node’s vicinity due to adding, deleting and loss of nodes, etc.). Thus, we propose to explore the resilence approach for ensuring the expected Quality of Service (QoS) after a disturbance. Resilience that is based on the viability framework, is the capacity of a system to undergo disturbance and maintain its functions and controls in the set of viability constraints [37]. We also intend to implement our operating policies derived from the viability and resilience approaches on a real WSN platform.

## Supporting information

S1 File The kernel viability in the 3-dimensional space. (PDF)
