# Supplementary material for: Adaptive management of energy consumption, reliability and delay of wireless sensor node: Application to IEEE 802.15.4 wireless sensor node
Source: PLoS One. 2017 Feb 24;12(2):e0172336. doi: 10.1371/journal.pone.0172336 (PMC5325272; doi:10.1371/journal.pone.0172336)
Supplement: S1 File — (PDF) [file pone.0172336.s001.pdf]

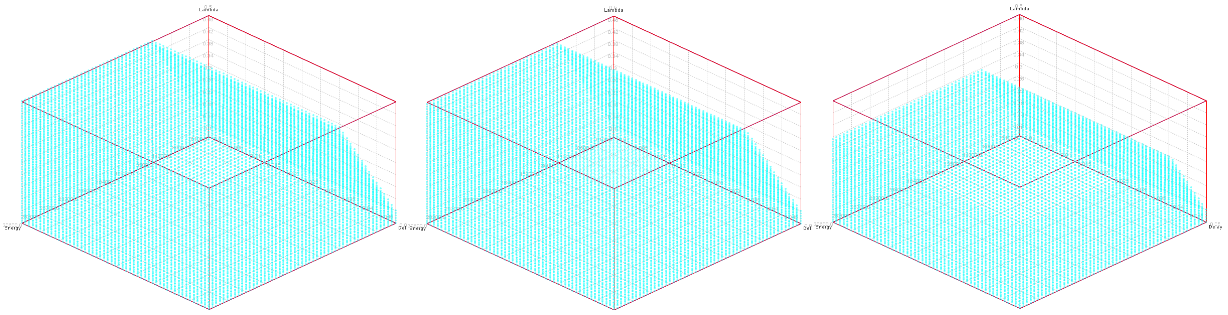

(a-1) R_{min} = 90% (a-2) R_{min} = 95% (a-3) R_{min} = 99%

(a) E_{sol} = 4 μJ/slot

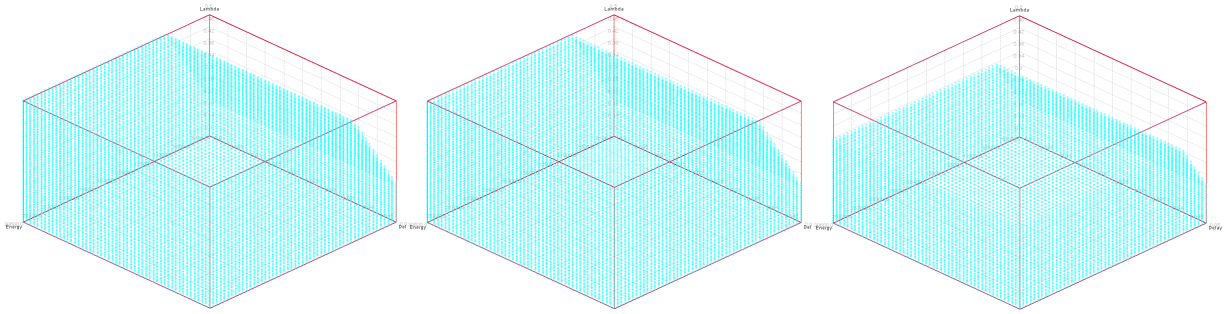

(b-1) R_{min} = 90% (b-2) R_{min} = 95% (b-3) R_{min} = 99%

(b) E_{sol} = 6 μJ/slot

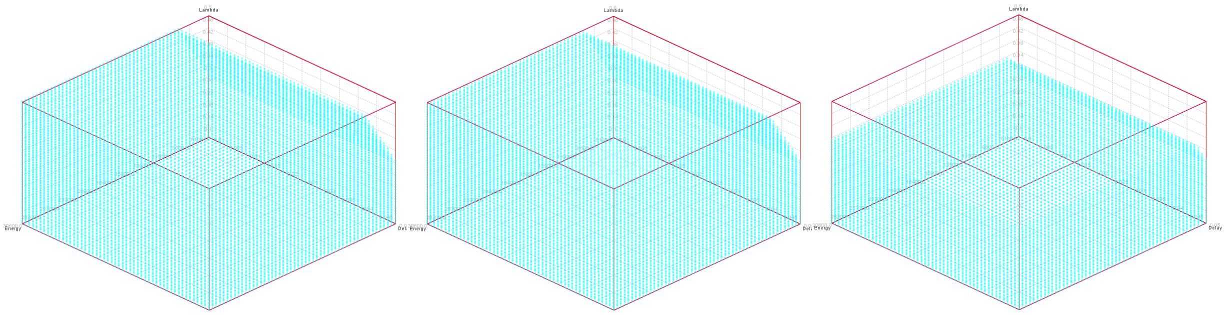

(c-1) R_{min} = 90% (c-2) R_{min} = 95% (c-3) R_{min} = 99%

(c) E_{sol} = 8 μJ/slot

Fig. A: Viability kernels from R_{min} of four-dimensional viability problem describing in Eqs. 23 to 26 by using values D_{max} = 50 ms, R_{min} = $\{0.9; 0.95; 0.99\}$, $VL_\lambda = 0.024$, $E_{sol} = \{4; 6; 8\}\,\mu$J/slot. The kernel viability is bordered by a green shape into the constraint space (defined by the cube with red edges) in the 3-dimensional space (E, D, λ)

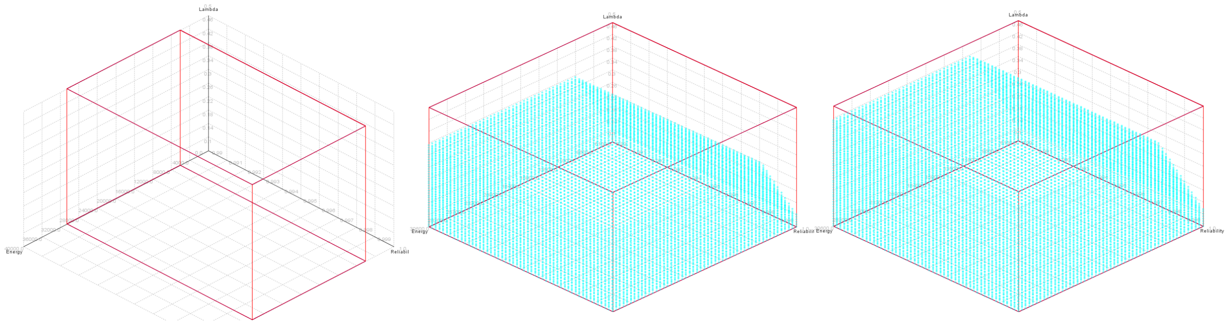

(a-1) D_{max} = 20 ms (a-2) D_{max} = 50 ms (a-3) D_{max} = 70 ms

(a) E_{sol} = 4 μJ/slot

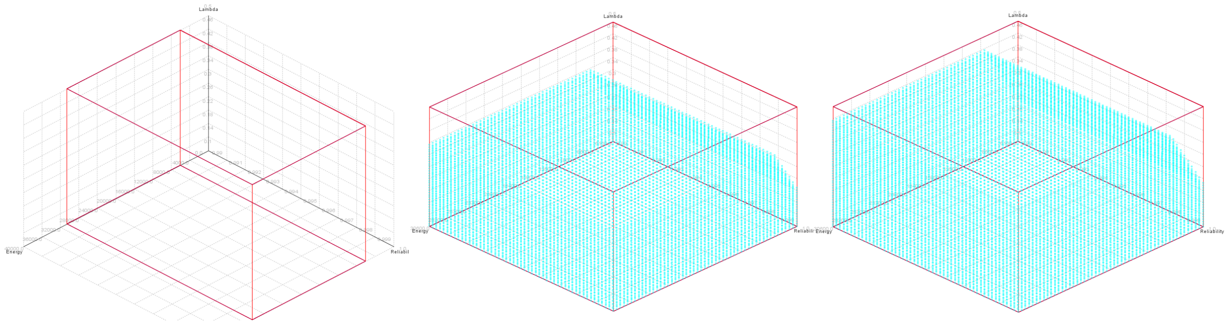

(b-1) D_{max} = 20 ms (b-2) D_{max} = 50 ms (b-3) D_{max} = 70 ms

(b) E_{sol} = 6 μJ/slot

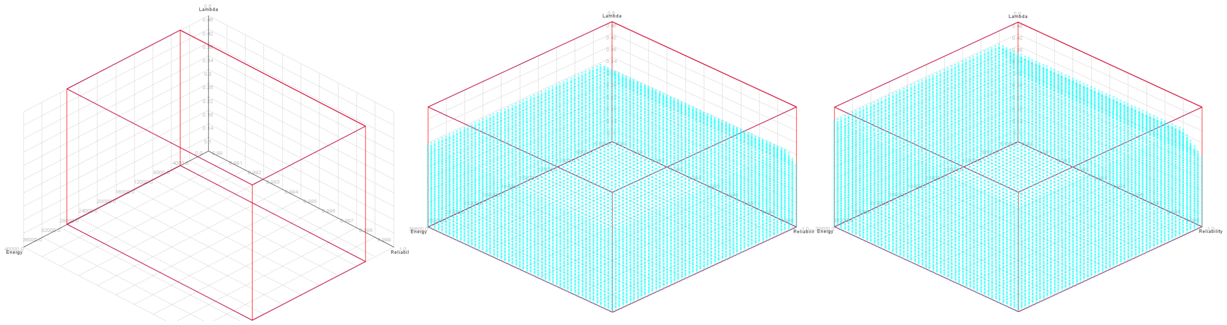

(c-1) D_{max} = 20 ms (c-2) D_{max} = 50 ms (c-3) D_{max} = 70 ms

(c) E_{sol} = 8 μJ/slot

Fig. B: Viability kernels from D_{max} of four-dimensional viability problem describing in Eqs. 23 to 26 by using values R_{min} = 99%, D_{max} = $\{20; 50; 70\}$ ms, VL_λ = 0.024, E_{sol} = $\{4; 6; 8\}$ μJ/slot. The kernel viability is bordered by a green shape into the constraint space (defined by the cube with red edges) in the 3-dimensional space (E, R, λ).
